# Real-world outcomes of immunotherapy in advanced NSCLC patients with comorbidities

**DOI:** 10.1038/s41416-026-03491-1

**Published:** 2026-06-08

**Authors:** HH Hektoen, KM Tsuruda, MT Mæhlen, Å Helland, BK Andreassen

**Affiliations:** 1 Department of Research, Cancer Registry of Norway, Norwegian Institute of Public Health, Oslo, Norway; 2 Department of Cancer Genetics, Institute for Cancer Research, Norwegian Radium Hospital, Oslo University Hospital, Oslo, Norway; 3 Department of Rheumatology, Oslo University Hospital, Oslo, Norway; 4 Division for Cancer Medicine, University of Oslo, Oslo, Norway

**Keywords:** Non-small-cell lung cancer, Cancer immunotherapy, Cancer epidemiology

## Abstract

**Background:**

Comorbidities are common in advanced non-small cell lung cancer (NSCLC) patients, yet their impact on systemic anti-cancer treatment (SACT) and survival in the era of immune checkpoint inhibitors (ICIs) remains unclear due to limited representation in clinical trials.

**Methods:**

This nationwide registry-based study included 9178 patients with stage IIIB–IV NSCLC diagnosed before and after ICI introduction. Kaplan–Meier and multivariable Cox models were applied to calculate hazard ratios (HRs) and 95% confidence intervals (CIs).

**Results:**

Common comorbidities included chronic obstructive pulmonary disease (COPD: 22%), cardiovascular disease (CVD: 21%), type 2 diabetes (T2D: 10%), and rheumatic and musculoskeletal diseases (RMD: 6%). Patients with comorbidities were less likely to receive SACT. Following ICI introduction, SACT use increased across all comorbidity groups and 2-year OS improved up to threefold. CVD patients had better 2-year OS when treated with ICI alone than ICI plus chemotherapy (38 vs 22%). Patients with CVD, T2D and RMD had higher risk of death when treated with ICI plus chemotherapy compared to patients without these comorbidities (HR = 1.36 (1.07–1.73), HR = 1.33 (0.99–1.78) and HR = 1.38 (0.97–1.95), respectively).

**Conclusions:**

Survival among patients with comorbidities improved following ICI introduction, however the benefit varied by comorbidity and treatment modality, supporting more individualised treatment strategies.

## Introduction

During the last decade, immune checkpoint inhibitors (ICIs) have transformed the treatment landscape for patients with advanced non-small cell lung cancer (NSCLC) without targetable mutations, offering improved survival over traditional platinum-based chemotherapy [1]. However, the benefits of ICIs are not universal and their effectiveness in more vulnerable populations, such as patients with coexisting diseases (comorbidities), remains poorly understood [2]. Patients with comorbidities are consistently underrepresented in clinical trials, limiting our knowledge of how comorbidities influence treatment response [3–5].

The majority of advanced lung cancer patients present with at least one comorbidity at diagnosis, and cardiovascular disease (CVD), chronic obstructive pulmonary disease (COPD), and type 2 diabetes (T2D) are among the most prevalent conditions [6–9]. Comorbidities can influence cancer treatment by increasing the risk of treatment-related complications, reducing treatment tolerance, and lowering the likelihood of receiving guideline-recommended therapies [10–12]. Moreover, the type and severity of comorbidity and the specific cancer therapy administered likely influence treatment outcomes [11]. However, the literature remains inconsistent regarding how comorbidities impact treatment patterns and clinical outcomes, particularly in the era of ICIs.

The tolerance of cancer treatments in patients with comorbidities can vary significantly between ICIs and chemotherapy, as these treatments differ markedly in their mechanisms of action and toxicity profiles [13]. ICIs work by activating the immune system against cancer, which can trigger immune-related adverse events [14]. Consequently, patients with autoimmune disorders face a higher risk of these events, which can lead to poorer outcomes [15, 16]. In contrast, chemotherapy kills rapidly dividing cells, including healthy ones, which can damage vital organs. As a result, patients with pre-existing organ dysfunction are more susceptible to adverse treatment effects from chemotherapy [17]. A well-documented example is the increased risk of chemotherapy-induced cardiotoxicity observed in patients with CVD [18, 19].

Current treatment guidelines provide limited direction on the use of ICIs in patients diagnosed with advanced NSCLC who have comorbidities. An exception is patients with active autoimmune disorders, for whom treatment is often approached with caution due to concerns about immune-related toxicities; nonetheless, there are no absolute contraindications to receiving ICI therapy [20, 21]. Many patients with comorbidities have been excluded from pivotal clinical trials and there is a substantial gap in evidence to guide treatment decisions for this large and clinically relevant patient group. Real-world data are critically needed to evaluate the impact of comorbidities on treatment delivery and outcomes in ICI-treated populations in clinical practice [2]. Few observational studies have addressed this question using large, population-based cohorts, and there has been even less focus on whether comorbidities differentially influence outcomes across treatment modalities [11, 22].

In this nationwide, population-based study from Norway, we aimed to investigate how comorbidities influence treatment patterns and survival outcomes in patients with advanced NSCLC. Specifically, we assessed how the introduction of ICIs has changed treatment patterns and examined whether the presence of comorbidities differentially affected outcomes in patients treated with ICIs versus chemotherapy. This evidence may inform personalized and more effective treatment strategies for patients with advanced NSCLC and comorbidities.

## Methods

### Study population

This retrospective cohort study included all individuals diagnosed with advanced (unresectable stage IIIB-IV) NSCLC in Norway during 2012–2021 and followed through June 30, 2023. Lung cancer cases were identified using International Classification of Diseases, 10th Revision (ICD-10) code C34, and tumor stage was defined according to the 8th edition of the AJCC TNM staging system. Histology subtypes were classified according to International Classification of Disease for Oncology 3rd Edition. Patients with small-cell carcinoma were excluded based on morphology codes 8041/4, 8043/3, 8045/3, leaving only NSCLC cases. Patients with targetable mutations were excluded and identified based on known epidermal growth factor receptor (EGFR) mutations or anaplastic lymphoma kinase (ALK) rearrangements, or information about receiving targeted treatment. Patients receiving treatment with a curative intention were excluded. This included surgery or curative radiotherapy with chemotherapy and/or durvalumab. We also excluded patients with other cancer history (up to two) years prior to diagnosis of NSCLC. A flow chart showing this selection is presented in Fig. S1.

Analyses were performed on the *overall cohort* that included all eligible patients as defined above, irrespective of palliative treatment received (if any), and on the *SACT cohort* that only included, patients treated with SACT in accordance with national guidelines.

### Data sources

This study was based on data from five nationwide, population-based registries in Norway: The Cancer Registry of Norway (CRN), the Norwegian Patient Registry (NPR), the Norwegian Prescription Database (NorPD), the Norwegian Cause of Death Registry, and Statistics Norway. These registries were linked at the individual level using the unique personal identification number assigned to all residents of Norway.

The CRN provided information on patient demographics and clinical characteristics related to cancer diagnoses. Owing to mandatory reporting, the registry has an estimated completeness of 99% for lung cancer cases [23]. Detailed data on systemic anticancer treatments (SACT) were sourced from the INSPIRE database (part of the CRN). This data covered nearly 90% of patients treated in Norway in 2019 [24]. Additional treatment-related information was retrieved from the NPR, which is an administrative database covering all patients who receive specialist healthcare in Norway, whether in hospitals, outpatient clinics, or from contracted specialists [25].

Data on comorbidities were derived from the NPR and NorPD. Diagnoses were coded using the ICD-10 and International Classification of Primary Care (2nd edition: ICPC-2) classification systems. NorPD contains individual-level information on all prescriptions dispensed at community pharmacies in Norway, classified using the Anatomical Therapeutic Chemical (ATC) system. It also includes ICD-10 and ICPC-2 codes specifying the indication for drug use.

Information about highest obtained educational level was obtained from Statistics Norway, and information on vital status and date of death from the Cause of Death Registry.

### First-line SACT

First-line SACT was defined as the initial systemic treatment administered after diagnosis of advanced disease. Treatment was categorized according to the period before and after the implementation of ICIs in Norwegian national guidelines (pre-ICI era: 2012–2016; post-ICI era: 2017–2021). In the pre-ICI era, standard first-line treatment according to Norwegian guidelines consisted of platinum-based doublet chemotherapy (hereafter referred to as *pre-ICI chemotherapy*). In the post-ICI era. *ICI monotherapy* (pembrolizumab) was implemented as standard first-line treatment for patients with tumour programmed death-ligand 1(PD-L1) expression ≥50% and no targetable driver mutations. From 2019, *ICI in combination with chemotherapy* (pembrolizumab in combination with platinum-based chemotherapy) was implemented for patients with non-squamous histology, regardless of PD-L1 expression. From 2020, ICI in combination with chemotherapy was extended to patients with squamous histology, independent of PD-L1 expression.

Patients were classified as receiving ICI in combination with chemotherapy if they received chemotherapy within ±6 days of their first pembrolizumab dose. First-line ICI treatment duration was defined as the number of days from the first recorded ICI dose to the last ICI dose administered before a change in systemic treatment type (including changes from monotherapy to combination therapy or vice versa). For patients who did not change treatment type, duration was calculated from the first to the last recorded ICI dose. If a treatment break exceeded 120 days, treatment duration was defined as the time from the first ICI dose to the last dose administered before the break.

### Comorbidities

We focused on the most prevalent comorbidities in our study population, including CVD, COPD, T2D and rheumatic and musculoskeletal disorders (RMD). Comorbidities were identified using ICD-10 diagnosis codes and corresponding treatment information (ATC codes) obtained from the NPR and the NorPD during the two years preceding the diagnosis of NSCLC. To ensure clinical relevance, a comorbidity was counted only if the patient had evidence of both a relevant hospital contact in the NPR and a corresponding prescription in the NorPD, or a prescription in the NorPD linked to the relevant diagnosis.

The Charlson Comorbidity Index (CCI) [26] was calculated using ICD-10 codes from inpatient and outpatient diagnoses recorded in the NPR within the 2 year period prior to the NSCLC diagnosis. Cancer and metastatic disease were excluded from the CCI calculation. The selection of the most prevalent comorbidities included in this study were based on the most frequent comorbidities identified by the conditions in the CCI. A more detailed description of the definition of the comorbidities and the disease codes included in the CCI index is shown in Table S1.

### Covariates

We used the following information from the time of diagnosis: age (years); sex (male, female); histology (adenocarcinoma, squamous cell, other); clinical stage (IIIB, IIIC, IV); Eastern Cooperative Oncology Group performance status (ECOG PS: 0, 1, 2, 3, 4); PD-L1 expression level ( < 1%, 1–49%, 50–74% and ≥75%), multiple comorbidities (0, 1, or >1); mutation status (EGFR/ALK). We also included information on vital status and date of death, emigration, or last follow-up; and information on radiotherapy (intention to treat, region codes). Palliative radiation was defined using the intention to treat information registered in the radiation information system by the oncologist. It was not included when defining treatment lines. Lastly, we included information on highest obtained educational level (less than college/university, college/university).

### Statistical analysis

Baseline patient and clinical characteristics were presented using descriptive statistics, for the overall cohort and stratified by type of comorbidity. Medians and interquartile ranges (IQR) or ranges were reported for continuous variables. Frequencies and percentages were presented otherwise. First line treatment patterns were illustrated separately for the pre-ICI (2012–16) and post-ICI eras (2017–2019) and stratified by comorbidity. Differences in the proportions of patients receiving SACT between the pre-ICI and post-ICI periods were evaluated using Pearson’s chi-square test.

The primary outcome was overall survival (OS). Patients were followed from diagnosis until death from any cause when studying the overall cohort (including those not receiving SACT) or from initiation of first line treatment when studying SACT cohort. Follow-up time was censored for all patients at the time of emigration or the end of the study period (June 30, 2023). Median follow-up time was calculated using the reverse Kaplan-Meier estimator [27].

Unadjusted OS was estimated using the Kaplan-Meier method and stratified by comorbidity groups. We reported 6 month and 2-year unadjusted OS and the ratio between the two. We also applied multivariable Cox proportional hazards regression models for each comorbidity group to estimate adjusted hazard ratios (HRs) with 95% confidence intervals (CIs) illustrating the association between comorbidity and risk of all-cause death among patients in the SACT cohort. Hazard ratios reflected average effects over time. We created separate models for each treatment group (pre-ICI chemotherapy, ICI monotherapy, ICI plus chemotherapy) stratified by comorbidity group. All models were adjusted for age at diagnosis, sex, histology, educational level, disease stage, and the presence of multiple comorbidities. We included unknown as a category in the analysis if data were missing. For patients treated with ICI, we additionally adjusted for ECOG PS and PD-L1 expression. A sensitivity analysis was performed on the adjusted models, restricting our study population to patients under 80 years of age and with an ECOG PS < 2 to elucidate whether observed effects were due to frailty or the comorbidity of interest (Fig. S2).

All analyses were conducted using Stata version 19.5 (StataCorp, College Station, TX).

## Results

**Fig. 1:**
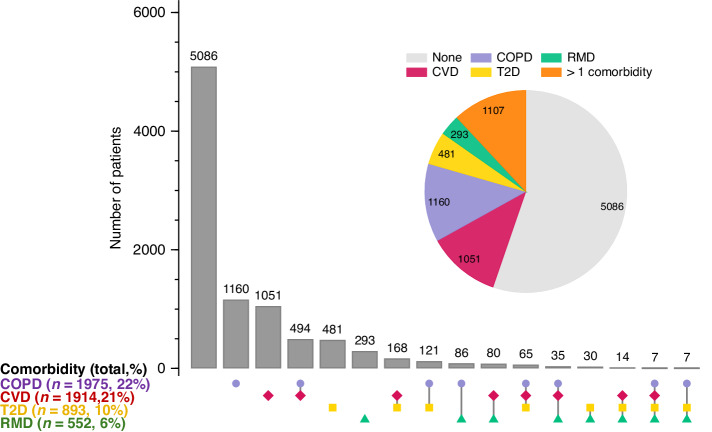
Prevalence of the most common comorbidities among patients in the overall cohort diagnosed with advanced (stage IIIB-IV) NSCLC (C34) in Norway between 2012 and 2021. Comorbidities occurred in the two years prior to diagnosis. The pie chart illustrates the distribution of comorbidities observed in our patient population and the upset plot illustrates the observed combinations of comorbidities. CVD cardiovascular disease, COPD chronic obstructive pulmonary disease, T2D Type 2 diabetes, RMD rheumatic and musculoskeletal disorders.

The overall cohort included 9178 patients diagnosed with advanced NSCLC in Norway without targetable mutations between 2012 and 2021, of whom 4672 received first line systemic treatment in accordance with national guidelines (SACT cohort). Median follow-up time was 96 and 42 months for patients diagnosed in the pre- and post-ICI eras, respectively. 45% of patients required medical treatment or hospital contact within the two years preceding their NSCLC diagnosis for at least one of the comorbidities examined. COPD was the most prevalent comorbidity (22%), followed by CVD (21%), T2D (10%), and RMD (6%). Many patients had two or more comorbidities, with the most frequent combinations being CVD and COPD (*n* = 494, 6%), and CVD and TD2 (*n* = 168, 2%) (Fig. 1).

### Changes in treatment patterns and survival following ICI introduction (overall cohort)

#### Baseline Characteristics

Individuals with comorbidities were slightly older at diagnosis than observed in the overall cohort (i.e. all NSCLC cases, including patients with comorbidities): the median age at diagnosis ranged from 70–71 years across the four comorbidity groups but was 68 years in the overall cohort (Table 1). A higher proportion of males was observed among patients with CVD (63%) and T2D (65%) compared to the overall cohort (56% males). In contrast, the proportion of males was lower among patients with RMD (43%). Patients with comorbidities, particularly those with COPD, had a higher prevalence of squamous cell carcinoma (30%) compared to the overall cohort (22%).

**Table 1:** Patient characteristics stratified by comorbidity, frequencies and percentages are reported unless otherwise specified.

	Overall cohort	COPD	CVD	T2D	RMD
	*N* = 9178	*N* = 1975	*N* = 1914	*N* = 893	*N* = 552
Age, median [range]	68 [22, 93]	70 [46, 89]	71 [46, 88]	70 [47, 90]	70 [43, 92]
Age, categories					
<65 years	2380 (26%)	333 (17%)	252 (13%)	168 (19%)	97 (18%)
65-74 years	3743 (41%)	880 (45%)	750 (39%)	395 (44%)	233 (42%)
≥75 years	3055 (33%)	762 (39%)	912 (48%)	330 (37%)	222 (40%)
Sex					
Females	4025 (44%)	883 (45%)	707 (37%)	316 (35%)	316 (57%)
Males	5153 (56%)	1092 (55%)	1207 (63%)	577 (65%)	236 (43%)
ECOG PS					
ECOG 0-1	3174 (35%)	596 (30%)	519 (27%)	280 (31%)	176 (32%)
ECOG 2-4	2370 (26%)	607 (31%)	618 (32%)	283 (32%)	162 (29%)
Unknown	3634 (40%)	772 (39%)	777 (41%)	330 (37%)	214 (39%)
PD-L1 expression					
<1% (incl 0%)	1178 (13%)	261 (13%)	211 (11%)	119 (13%)	92 (17%)
1-49%	1047 (11%)	221 (11%)	211 (11%)	120 (13%)	67 (12%)
50-74%	631 (7%)	133 (7%)	126 (7%)	55 (6%)	39 (7%)
≥75%	790 (9%)	143 (7%)	140 (7%)	74 (8%)	37 (7%)
Unknown	5532 (60%)	1217 (62%)	1226 (64%)	525 (59%)	317 (57%)
Stage					
IIIB/C	1250 (14%)	349 (18%)	261 (14%)	128 (14%)	72 (13%)
IV	7928 (86%)	1626 (82%)	1653 (86%)	765 (86%)	480 (87%)
Histology					
Squamous	2036 (22%)	587 (30%)	457 (24%)	231 (26%)	140 (25%)
Non-squamous	7142 (78%)	1388 (70%)	1457 (76%)	662 (74%)	412 (75%)
Radiation against brain					
No	8113 (88%)	1801 (91%)	1788 (93%)	810 (91%)	509 (92%)
Yes	1065 (12%)	174 (9%)	126 (7%)	83 (9%)	43 (8%)
Cancer treatment					
SACT According to guidelines	4672 (51%)	888 (45%)	728 (38%)	368 (41%)	230 (42%)
Other SACT	343 (4%)	62 (3%)	63 (3%)	39 (4%)	19 (3%)
NO SACT Only radiation	1650 (18%)	419 (21%)	381 (20%)	197 (22%)	135 (24%)
No recorded treatment	2513 (27%)	606 (31%)	742 (39%)	289 (32%)	168 (30%)
Number of comorbidities^a^					
None	5086 (55%)	-	-	-	-
Only one	2985 (33%)	1160 (59%)	1051 (55%)	481 (54%)	293 (53%)
More than one	1107 (12%)	815 (41%)	863 (45%)	412 (46%)	259 (47%)
Charlson comorbidity index					
CCI 0	5395 (59%)	532 (27%)	237 (12%)	169 (19%)	179 (32%)
CCI 1-2	3330 (36%)	1260 (64%)	1333 (70%)	540 (60%)	320 (58%)
CCI ≥ 3	453 (5%)	183 (9%)	344 (18%)	184 (21%)	53 (10%)
Highest obtained education					
Vocational school, high school, or lower	8014 (87%)	1803 (91%)	1695 (89%)	801 (90%)	494 (89%)
College or university	1087 (12%)	161 (8%)	204 (11%)	84 (9%)	57 (10%)
Unknown	77 (1%)	11 (1%)	15 (1%)	8 (1%)	1 (0%)

*ECOG PS* Eastern Cooperative Oncology Group performance status, *PD-L1* Programmed death-ligand 1, *CVD* cardiovascular disease, *COPD* chronic obstructive pulmonary disease, *T2D* Type 2 diabetes, *RMD* rheumatic and musculoskeletal disorder.

^a^Number of comorbidities among the selected in this study: CVD, COPD, T2D, or RMD.

#### Treatment patterns

In the overall cohort, the proportion of patients receiving any SACT increased from 52% to 57% after the introduction of ICIs (Table S2). Within each comorbidity group, the proportion of patients receiving SACT increased from pre- to post-ICI: COPD (45% to 51%), CVD (38% to 45%), T2D diabetes (43% to 48%) and RMD (40% to 50%).

**Fig. 2:**
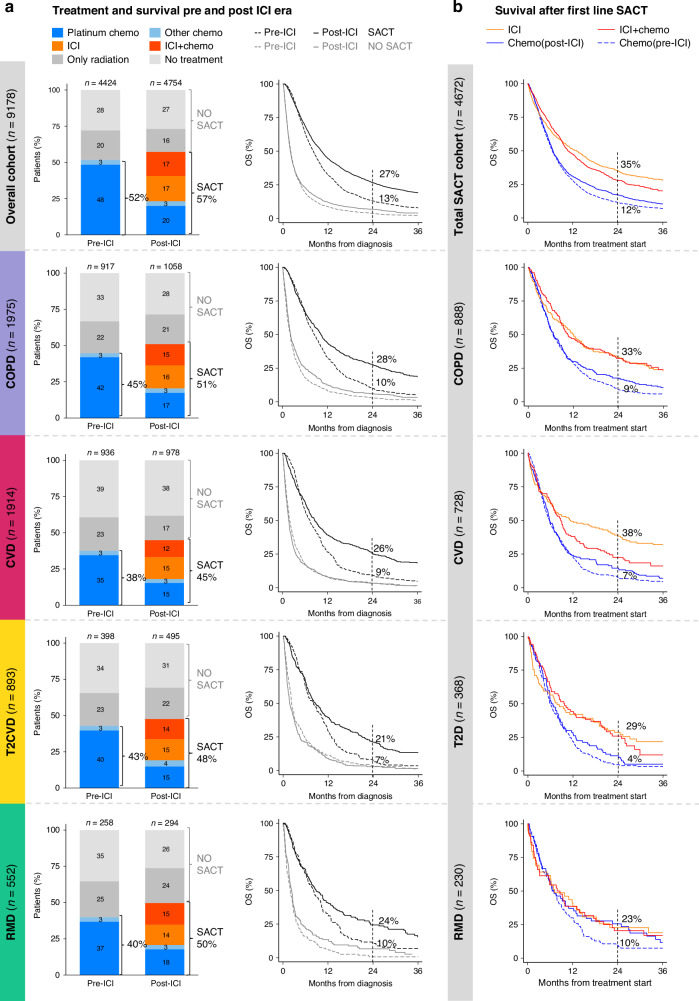
Trends in systemic anti-cancer treatment (SACT) and overall survival (OS) pre (2012–16) and post (2017–21) immune checkpoint inhibitors (ICIs) were implemented. **a** Trends in treatment and survival pre- and post-ICI era, including patients who did not recieve SACT (no SACT). Results are shown for the overall cohort and stratified by comorbidity group (COPD, CVD, type 2 diabetes, and RMD). Twenty-four-month OS estimates are shown for SACT treated patients pre- and post-ICI era. **b** OS among SACT treated patients, stratified by systemic treatment type. Results are shown for the total SACT cohort and by each comorbidity group within the SACT cohort. Twenty-four-month OS estimates are shown for patients treated with chemotherapy pre-ICI era (Chemo pre-ICI) and those treated with ICI. CVD cardiovascular disease, COPD chronic obstructive pulmonary disease, T2D Type 2 diabetes, RMD rheumatic and musculoskeletal disorders.

Examining specific SACT patterns in the overall cohort pre- and post-ICI introduction revealed similar treatment patterns across all comorbidity groups (Fig. 2a). In the pre-ICI period, most patients in the overall cohort received platinum-based chemotherapy as their first-line treatment (48%, of all diagnosed). Only a small proportion (3%) received non-platinum-based chemotherapy. During the post-ICI period, a similar proportion of patients in the overall cohort received ICI monotherapy and combination therapy (17% each). Among patients with CVD, a slightly higher proportion received monotherapy than combination therapy (15% vs 12%) For patients with RMD there was a tendency toward the opposite and slightly fewer patients received monotherapy than combination therapy (14% vs 15%).

#### Survival

A substantial improvement in survival was observed in the overall cohort across all comorbidity groups following the introduction of ICIs among patients receiving SACT. (Fig. 2a and Table S3). No notable differences were observed in short-term survival (6 months), indicating that the survival gains were primarily driven by improvements in long-term survival outcomes. In the overall cohort, two-year survival among SACT recipients increased 2.1-fold from the pre- to post-ICI period. Within comorbidity groups, the relative improvements were even greater. The most pronounced benefits were seen in patients with T2D (3.0-fold increase), CVD (2.9-fold), and COPD (2.8-fold). Patients with RMD experienced a 2.4-fold improvement in two-year survival. Patients who did not receive SACT did not experience any notable survival benefit after the introduction of ICIs.

### How patients with comorbidities responded to different SACT regimens compared to patients without these conditions (SACT cohort)

#### Baseline Characteristics—by treatment type and comorbidity groups

Among patients in the total SACT cohort (e.g. all patients treated with SACT in accordance with national guidelines), patients treated with ICI (with/without chemotherapy) were generally older than those treated with chemotherapy in the pre-ICI era (Table 2). Across comorbidity groups, the median age ranged from 68–70 years for patients receiving chemotherapy (pre-ICI) to 71–74 years among those receiving ICI monotherapy and 70–72 years among those receiving ICI in combination with chemotherpy.

**Table 2:** Patient characteristics stratified by comorbidity and treatment groups, chemo (pre-ICI), ICI monotherapy and ICI plus chemotherapy.

	Total SACT cohort			COPD			CVD			T2D			RMD		
	Chemo (pre-ICI)	ICI mono	ICI + chemo	Chemo (pre-ICI)	ICI mono	ICI + chemo	Chemo (pre-ICI)	ICI mono	ICI + chemo	Chemo (pre-ICI)	ICI mono	ICI + chemo	Chemo (pre-ICI)	ICI mono	ICI + chemo
	*N* = 2090	*N* = 794	*N* = 790	*N* = 373	*N* = 164	*N* = 154	*N *= 315	*N* = 141	*N* = 113	*N* = 150	*N* = 69	*N* = 69	*N* = 93	*N* = 39	*N* = 44
Age, median [range]	67 [28, 92]	70 [41, 93]	69 [36, 87]	69 [46, 87]	72 [49, 89]	70 [47, 86]	70 [46, 88]	72 [50, 88]	72 [49, 87]	69 [47, 86]	71 [53, 90]	70 [49, 83]	68 [43, 92]	74 [55, 85]	70 [55, 83]
Days to treatment, median[IQR]	27 [15, 48]	28 [18, 47]	27 [18, 40]	25[15, 43]	27 [16, 49]	27 [20, 43]	27 [14, 48]	30 [20, 49]	28 [19, 39]	27 [15, 49]	28 [18, 44]	30 [20, 44]	23 [13, 48]	29 [16, 46]	28 [22, 46]
Days on 1st line ICI, median[IQR]	-	101 [22, 295]	70 [23, 148]	-	89 [22, 257]	71 [22, 133]	-	78 [16, 212]	64 [18, 112]	-	43 [1, 232]	64 [22, 144]	-	43 [1, 174]	54 [1, 144]
**Sex**															
Females	896 (43%)	395 (50%)	366 (46%)	150 (40%)	85 (52%)	76 (49%)	112 (36%)	49 (35%)	47 (42%)	50 (33%)	27 (39%)	23 (33%)	50 (54%)	26 (67%)	22 (50%)
Males	1194 (57%)	399 (50%)	424 (54%)	223 (60%)	79 (48%)	78 (51%)	203 (64%)	92 (65%)	66 (58%)	100 (67%)	42 (61%)	46 (67%)	43 (46%)	13 (33%)	22 (50%)
**ECOG PS**															
ECOG 0-1	565 (27%)	506 (64%)	570 (72%)	99 (27%)	90 (55%)	99 (64%)	81 (26%)	81 (57%)	86 (76%)	44 (29%)	42 (61%)	48 (70%)	24 (26%)	22 (56%)	30 (68%)
ECOG 2-4	143 (7%)	214 (27%)	169 (21%)	36 (10%)	52 (32%)	42 (27%)	29 (9%)	46 (33%)	22 (19%)	11 (7%)	18 (26%)	14 (20%)	10 (11%)	12 (31%)	10 (23%)
Unknown	1382 (66%)	74 (9%)	51 (6%)	238 (64%)	22 (13%)	13 (8%)	205 (65%)	14 (10%)	5 (4%)	95 (63%)	9 (13%)	7 (10%)	59 (63%)	5 (13%)	4 (9%)
**Stage**															
IIIB/C	269 (13%)	133 (17%)	76 (10%)	56 (15%)	42 (26%)	17 (11%)	46 (15%)	26 (18%)	9 (8%)	11 (7%)	9 (13%)	9 (13%)	14 (15%)	3 (8%)	3 (7%)
IV	1821 (87%)	661 (83%)	714 (90%)	317 (85%)	122 (74%)	137 (89%)	269 (85%)	115 (82%)	104 (92%)	139 (93%)	60 (87%)	60 (87%)	79 (85%)	36 (92%)	41 (93%)
**Histology**															
Squamous	393 (19%)	177 (22%)	108 (14%)	98 (26%)	46 (28%)	30 (19%)	69 (22%)	36 (26%)	20 (18%)	32 (21%)	19 (28%)	11 (16%)	17 (18%)	6 (15%)	6 (14%)
Non-squamous	1697 (81%)	617 (78%)	682 (86%)	275 (74%)	118 (72%)	124 (81%)	246 (78%)	105 (74%)	93 (82%)	118 (79%)	50 (72%)	58 (84%)	76 (82%)	33 (85%)	38 (86%)
**PD-L1**															
<50%	116 (6%)	46 (6%)	459 (58%)	13 (3%)	10 (6%)	94 (61%)	9 (3%)	13 (9%)	69 (61%)	11 (7%)	6 (9%)	41 (59%)	5 (5%)	6 (15%)	26 (59%)
≥50%	60 (3%)	689 (87%)	142 (18%)	8 (2%)	139 (85%)	21 (14%)	5 (2%)	111 (79%)	17 (15%)	6 (4%)	58 (84%)	8 (12%)	4 (4%)	32 (82%)	9 (20%)
Unknown result	1914 (92%)	59 (7%)	189 (24%)	352 (94%)	15 (9%)	39 (25%)	301 (96%)	17 (12%)	27 (24%)	133 (89%)	5 (7%)	20 (29%)	84 (90%)	1 (3%)	9 (20%)
**Radiation, brain**															
No	1779 (85%)	667 (84%)	697 (88%)	340 (91%)	144 (88%)	140 (91%)	286 (91%)	130 (92%)	102 (90%)	125 (83%)	62 (90%)	62 (90%)	85 (91%)	35 (90%)	40 (91%)
Yes	311 (15%)	127 (16%)	93 (12%)	33 (9%)	20 (12%)	14 (9%)	29 (9%)	11 (8%)	11 (10%)	25 (17%)	7 (10%)	7 (10%)	8 (9%)	4 (10%)	4 (9%)
Number of Comorbidities*															
None	1348 (64%)	461 (58%)	481 (61%)												
Only one	572 (27%)	263 (33%)	245 (31%)	243 (65%)	113 (69%)	107 (69%)	187 (59%)	85 (60%)	60 (53%)	89 (59%)	43 (62%)	48 (70%)	53 (57%)	22 (56%)	30 (68%)
More than one	170 (8%)	70 (9%)	64 (8%)	130 (35%)	51 (31%)	47 (31%)	128 (41%)	56 (40%)	53 (47%)	61 (41%)	26 (38%)	21 (30%)	40 (43%)	17 (44%)	14 (32%)
**Highest obtained education**															
Vocational school, high school, or lower	1824 (87%)	681 (86%)	656 (83%)	349 (94%)	143 (87%)	129 (84%)	279 (89%)	120 (85%)	95 (84%)	136 (91%)	57 (83%)	58 (84%)	79 (85%)	33 (85%)	36 (82%)
College or university	247 (12%)	109 (14%)	125 (16%)	22 (6%)	18 (11%)	23 (15%)	34 (11%)	20 (14%)	16 (14%)	11 (7%)	12 (17%)	11 (16%)	13 (14%)	6 (15%)	8 (18%)
Unknown	19 (1%)	4 (1%)	9 (1%)	2 (1%)	3 (2%)	2 (1%)	2 (1%)	1 (1%)	2 (2%)	3 (2%)	0 (0%)	0 (0%)	1 (1%)	0 (0%)	0 (0%)

Frequencies and percentages are reported unless otherwise specified.

*ECOG PS* Eastern Cooperative Oncology Group performance status, *PD-L1* Programmed death-ligand 1, *CVD* cardiovascular disease, *COPD* chronic obstructive pulmonary disease, *T2D* type 2 diabetes, *RMD* rheumatic and musculoskeletal disorders, *ICI* Immune checkpoint inhibitors, *mono* monotherapy, *Chem*o Chemotherapy.

*Number of comorbidities among the selected in this study: CVD, COPD, T2D, or RMD.

Overall, the pattern in characteristics among patients receiving ICI as monotherapy versus in combination with chemotherapy was similar across comorbidity groups. In general, patients treated with ICI monotherapy were older and more likely to have a higher ECOG performance status compared with those receiving combination therapy. Patients with CVD and COPD were those with the highest proportion of ECOG-2 patients receiving ICI monotherapy. Patients with COPD also had a higher proportion of ECOG-2 patients receiving ICI in combination with chemotherapy 27% vs 21% in the total SACT cohort.

The largest variations between comorbidity groups related to patient sex. In the total SACT cohort, male patients made up 50% of those treated with ICI monotherapy and 54% of those treated with ICI in combination with chemotherapy. Among patients with CVD, 65% of those treated with ICI monotherapy and 58% of those treated with combination therapy were male. In contrast, only 33% of patients with RMD receiving ICI monotherapy and 50% of those receiving combination therapy were male.

There were also variations in the duration of first-line ICI treatment. In the total SACT cohort, the median duration of ICI monotherapy was 101 days, compared with 89 days for patients with COPD, 78 days for those with CVD, and 43 days for patients with T2D and RMD (Table 2).

#### Survival outcomes by treatment type and comorbidity groups

Within the total SACT cohort, ICI monotherapy and ICI in combination with chemotherapy were associated with substantial increases in two-year OS (35% and 28%, respectively), compared with chemotherapy alone in the pre- and post- eras (12% and 17%, respectively; Fig. 2b and Table S4). Similar results were observed for patients with COPD, who experienced notable improvements in 2-year OS for ICI monotherapy (33%) and combination therapy (32%), compared with chemotherapy (pre-ICI: 9%, post-ICI: 17%).

Among patients with CVD, the greatest survival benefit was observed with ICI monotherapy (2-year OS: 38%), which exceeded that of ICI in combination with chemotherapy (22%) (Fig. 2c and Table S4). Both ICI approaches provided clear survival advantages over chemotherapy for these patients. Patients with T2D also experienced improved survival, with a two-year OS of 29% after first-line ICI monotherapy and 24% after combination therapy, compared with chemotherapy alone in the pre-ICI (4%) and post-ICI (8%) periods.

Patients with RMD showed the most distinct survival pattern post-ICI. In this group, 2-year OS post-ICI was similar across treatment modalities: 24% with chemotherapy, 23% with ICI monotherapy, and 20% with ICI in combination with chemotherapy. However, two-year survival during pre-ICI period was notably lower (10%).

#### Effect of comorbidity (vs not having comorbidity) on survival by treatment type

**Fig. 3:**
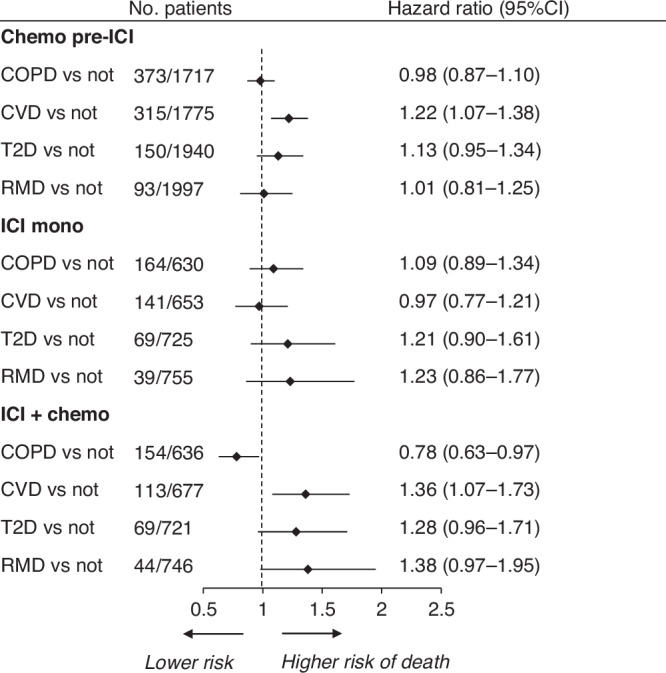
Risk of death from any cause among patients in the SACT cohort with a specific comorbidity vs those without that diagnosis, stratified by first-line systemic anticancer therapy. A hazard ratio of 1 indicates that the comorbidity does not increase the risk of death compared to patients without that comorbidity. All models were adjusted for sex, age, histology, education, stage and the presence of multiple (2 or more) comorbidities. Analyses related to first-line ICI treatment were also adjusted for PD-L1 expression and ECOG PS. ICI immune check point inhibitors, CVD cardiovascular disease, COPD chronic obstructive pulmonary disease, T2D Type 2 diabetes, RMD rheumatic and musculoskeletal disorders.

In the adjusted Cox regression models, the impact of comorbidities on survival varied across treatment type (Fig. 3). In the pre-ICI era when chemotherapy was the only option, patients with CVD had a significantly higher risk of death compared to those without CVD (HR 1.22, 95% CI 1.07–1.38), whereas COPD and RMD did not appear to influence survival.

For those treated with first line ICI monotherapy, CVD was not associated with increased mortality risk (HR 0.97, 95% CI 0.77–1.21). In contrast, patients with T2D and RMD showed a tendency toward higher risk of death. These associations were not statistically significant.

For patients treated with ICI in combination with chemotherapy, CVD was associated with a significantly increased risk of death (HR 1.36, 95% CI 1.07–1.73). Elevated risks were also observed for patients with T2D (HR 1.33, 95% CI 0.99–1.78) and RMD (HR 1.38, 95% CI 0.97–1.95), although these were not statistically significant. Patients with COPD treated with ICI in combination with chemotherapy had a significantly lower risk of death compared to patients without COPD undergoing the same treatment (HR 0.78, 95% CI 0.63–0.97).

Sensitivity analyses restricted to patients < 80 years at diagnosis and ECOG PS < 2 yielded results consistent with those described above (Fig. S2).

## Discussion

In this nationwide real-world study, we investigated the influence of common comorbidities (COPD, CVD, T2D, or RMD) on treatment patterns and survival outcomes among patients with advanced NSCLC before and after the introduction of ICI therapy. To our knowledge, this is the first comprehensive study to illustrate the effects of ICIs across the most common comorbidity groups and to compare outcomes to the pre-ICI era. We observed that a higher proportion of patients received SACT in the post ICI-era, including those with comorbidities, suggesting that ICIs have expanded treatment access for these patients since the pre-ICI era. Moreover, substantial and consistent improvements in OS were observed across all comorbidity groups after the introduction of ICIs, with relative survival gains that exceeded those observed in the overall cohort.

The survival increased among advanced NSCLC patients following the introduction of ICIs in clinical practice and is consistent with findings from randomized clinical trials and real-world studies demonstrating superior survival with ICIs compared to chemotherapy [28–30]. A novel contribution from our study is the demonstration that survival gains associated with ICIs were also pronounced among patients with certain comorbidities. Studies examining the effect of ICIs in patients with comorbidities remain scarce because these patients are frequently underrepresented in pivotal clinical trials [31]. A few real-world studies have shown that higher comorbidity burden negatively influences ICI outcome [32–34]. However, advanced NSCLC patients with comorbidities generally have a poorer prognosis irrespective of treatment and it can be challenging to disentangle the true effect of ICIs [9, 35]. By comparing survival within the same disease groups before and after the introduction of ICIs for NSCLC, we demonstrated that individuals with certain comorbidities, who previously had particularly poor outcomes with chemotherapy, experienced up to three-fold increase in two-year OS benefit, in which was even greater than the overall cohort that showed a 2-fold increase in OS following the introduction of ICIs. Importantly, we observed no substantial change in survival across the study periods among patients who remained untreated (no SACT). This strengthens the notion that the survival gains we observed are primarily attributable to the increased use and effectiveness of ICI therapies, rather than broader improvements in supportive care or diagnostic practices alone.

As observed in previous studies, patients with comorbidities were less likely to receive SACT than the overall study cohort [12, 36]. However, there was an increase in SACT use for patients with comorbidities following ICI introduction, probably due to the favorable toxicity profile and improved tolerability of ICIs compared with conventional cytotoxic chemotherapy [37]. The general increase in SACT over time could also be a result of clinicians having more available treatment options.

We also observed variation in first line treatment choice across comorbidity groups. Patients with CVD were more likely to receive ICI monotherapy rather than ICI in combination with chemotherapy. In contrast, patients with RMD showed a weak opposite trend, with a higher proportion receiving combination therapy. Moreover, patients with RMD were more frequently treated with chemotherapy post ICI era than those with CVD. These patterns suggest that clinicians tend to be more cautious when prescribing chemotherapy-based regimens for patients with CVD, but not for those with RMD. This aligns with previous findings indicating that clinicians often avoid chemotherapy in patients with CVD due to the elevated risk of cardiotoxicity [12]. In contrast, among patients with RMD, adding chemotherapy to ICI treatment may help maintain disease control of the RMD, which could partly explain the preference for combination therapy in this patient group.

Interestingly, the prognostic impact of comorbidities differed by treatment type. In the pre-ICI era, CVD was associated with increased mortality (compared to no diagnosis of CVD), even after adjusting for the most important prognostic factors. This finding is consistent with previous studies linking cardiovascular comorbidity to poor tolerance and higher toxicity from cytotoxic regimens [12]. Indeed, CVD was also associated with poorer outcomes in patients treated with ICI in combination with chemotherapy, highlighting the potential additive burden of cytotoxic treatment in this group. In contrast, when treated with ICI monotherapy, patients with CVD achieved survival comparable to those who did not have CVD. These favorable outcomes associated with ICI monotherapy, but not with ICI in combination with chemotherapy warrant further investigation. Given the overrepresentation of males among patients with CVD, it is tempting to speculate whether sex differences may contribute to these findings, even though we adjusted for sex. Previous studies have suggested that men derive relatively greater benefit from ICI monotherapy than from combination therapy compared with women, which mirrors the pattern observed in our CVD subgroup [38–40].

Patients with autoimmune diseases, including RMD, have been consistently excluded from ICI clinical trials due to concerns about immune related side-effects [41]. In our study, we observed improved survival among patients with RMD following the introduction of ICIs. However, this improvement was not independently associated with ICI-based treatment, as was observed for other comorbidity subgroups, but appeared to be driven also by increased survival among those treated with chemotherapy in the post-ICI era. Importantly, NSCLC patients with RMD did not show an increased risk of death when treated with chemotherapy compared to patients without RMD. In contrast, there was a tendency toward higher mortality among patients with RMD receiving ICIs compared to patients without RMD. While autoimmune comorbidity is associated with elevated risk of ICI-related toxicity, its impact on OS remains less clear. Few studies have reported outcomes comparable to patients without autoimmune disease [41–43]. Additionally, concomitant treatment with systemic corticosteroids or other immunosuppressants has been linked to reduced ICI efficacy [44]. In our cohort, only RMD patients who had received high-dose steroids or immunosuppressants were included, which may partly explain the poorer survival observed.

Patients with T2D showed a trend toward higher mortality after first-line ICI treatment compared with those without T2D. Several studies have reported reduced OS among ICI-treated patients with T2D, which may reflect diabetes-associated immune dysfunction or potential interactions with antidiabetic medications that attenuate ICI efficacy [45]. ICIs have also been reported to worsen coexisting T2D in cancer patients [46].

In our cohort, patients with coexisting T2D and RMD received shorter durations of both ICI monotherapy and ICI in combination with chemotherapy compared with other subgroups, which could be due to poorer OS or may reflect treatment discontinuation due to toxicity or immune-related adverse events. This should be looked further into in a larger study.

Patients with coexisting COPD experienced substantial survival benefit following the introduction of ICIs, with particularly high two-year survival observed among those treated with ICI-chemotherapy combinations compared with the overall cohort. After adjustment for key prognostic factors including age, sex, and performance status, coexisting COPD was associated with a lower risk of death compared to the absence of COPD. Prior studies suggest that COPD may enhance ICI responsiveness, possibly due to chronic inflammation leading to higher tumor mutational burden [47, 48]. However, in our cohort, this apparent benefit was limited to patients receiving combination therapy and not observed with ICI monotherapy. One explanation for this could be that COPD patients receiving ICI monotherapy had more severe COPD than those receiving combination therapy, and it is primarily those with a mild COPD that show favorable outcomes with ICIs [47].

Coexisting diseases influence cancer treatment and outcomes through multiple mechanisms, making it challenging to disentangle their true impact on ICI effectiveness. In the present study, this complexity is addressed by following the same patient groups over time and examining how the introduction of ICIs affected survival within these specific populations. We adjusted for key prognostic variables including performance status, age, sex, disease stage, and PD-L1 expression, to account for differences in baseline risk. Sensitivity analyses restricted to patients younger than 80 years with good performance status led to comparable results, suggesting that our findings are not solely explained by frailty. Nevertheless, additional analyses are needed to clarify whether the observed differences in ICI outcomes are primarily driven by the comorbidities themselves, and the extent to which concomitant medications, or other disease-specific factors modify treatment tolerance and efficacy.

Key strengths of this study include the use of high-quality population-based national registries, reflecting patients treated in real-world clinical practice. The availability of comprehensive information about NCSCL patients and their comorbidities across the periods before and after the introduction of ICIs allowed us to evaluate temporal changes in treatment patterns and survival among comparable patient populations. Several limitations must still be acknowledged. Since this is an observational study, patients included in our study received treatment based on their personal clinical scenario. Although we adjusted for factors that were likely to inform treatment decisions (e.g., age and performance status) we cannot rule out confounding from unavailable clinical factors such as tumor aggressiveness, smoking status, or patient preference. Other potential sources of unmeasured confounding are comorbidity severity, concomitant medications (eg, corticosteroids), and immune-related adverse events, which were not captured in our data. Additionally, PD-L1 expression and ECOG performance status were only available in the ICI era, potentially introducing imbalance in pre- versus post-ICI comparisons.

## Conclusion

Patients with advanced NSCLC and common comorbidities experienced improved overall survival following the introduction of ICIs, with relative gains exceeding those observed in the overall cohort, reflecting poorer outcomes among patients with comorbidities before the ICI era. These findings indicate that ICIs have expanded treatment opportunities for patients previously considered ineligible for SACT. Survival benefits, however, varied by comorbidity type and treatment regimen, underscoring the importance of more tailored therapeutic strategies based on individual comorbidity profiles. Further research is needed to optimise ICI use in these clinically diverse populations.

## Supplementary information

Supplementary

STROBE checklist

## Data Availability

The Cancer Registry of Norway’s data-delivery unit handles all inquiries about data from the Cancer Registry of Norway via application to Helsedata.no. Access will be conditional to adherence to local ethical and security policy. The STATA code used to conduct specific analyses is available from the authors after request.
