# Supplementary material for: Real-world outcomes of immunotherapy in advanced NSCLC patients with comorbidities
Source: Br J Cancer. 2026 Jun 8;135(5):763–72. doi: 10.1038/s41416-026-03491-1 (PMC13478398; doi:10.1038/s41416-026-03491-1)
Supplement: Supplementary file 1 [file 41416_2026_3491_MOESM1_ESM.docx]

**Supplementary materials**

**Defining the comorbid diseases**

Information about comorbid diseases was assessed using administrative data about hospital visits from Norwegian Patient Registry *(NPR)* and dispensed prescription drugs from Norwegian Prescribed Drug Registry (*NorPD*).

Patients were classified as having a recent history of comorbid condition during the last two years prior to the treatment initiation:

- had ≥ 1 hospital visits (in- or outpatient) with an ICD-10 code for the specific disease in *NPR* ***and*** were dispensed ≥ 1 prescription reimbursed with an ICD-10 or ICPC-2 code for the specific disease in *NorPD*

**OR**

were dispensed ≥ 1 prescription reimbursed **with** an ICD-10 or ICPC-2 code for the specific disease in *NorPD*

The selection of comorbidities included in this study was based on the disease groups represented in the Charlson Comorbidity Index, focusing on the four most frequently registered comorbidities in the Norwegian Patient Registry (NPR). Cardiovascular diseases were combined into a single category, and we focused specifically on type 2 diabetes and chronic obstructive pulmonary disease (COPD) within the diabetes and chronic pulmonary disease groups, respectively. These conditions were the dominant diagnoses within their categories, allowing for a cleaner and more homogeneous grouping for analysis.

**Table S1** illustrates the codes used in defining the different comorbidities from the two main sources, Norwegian patient registry and the Norwegian Prescribed Drug Registry

| Comorbidities  based on conditions in the Charlson comorbidity index |  | **Norwegian Patient**  **Registry (NPR)** | | **Norwegian Prescribed Drug Registry** | |
| --- | --- | --- | --- | --- | --- |
| **Name** | **Comorbidity group** | **In contact with hospital (ICD10 codes)** | **Prevalence NPR (%)** | **Medications (ATC-codes)** | **Reimbursement (ICD10, ICPC2)** |
| Myorcardial infarction | CVD | I21.x, I22.x, I25.2 | 5 % | - | **-** |
| Congestive heart failure | CVD | I09.9, I11.0, I13.0, I13.2, I25.5, I42.0, I42.5–I42.9, I43.x, I50.x, P29 | 5 % | B01 & C excluding(C01C, C05 and C10) | ICD10**:**I43, I50, I09, I11, I13, I25, I42, P29 ICPC2: K77 |
| Peripheral vascular disease | CVD | I70.x, I71.x, I73.1, I73.8, I73.9, I77.1, I79.0, I79.2, K55.1, K55.8, K55.9, Z95.8, Z95.9 | 7 % | B01 & C excluding(C01C, C05 and C10) | ICD10: I70, I71, I73, I77, I79, I79, K55, K55, K55, Z95 ICPC2: K92, K99 |
| Cerebrovascular disease | CVD | G45.x, G46.x, H34.0, I60.x–I69.x | 5 % | - | - |
| Diabetes (type 2)  With/without complications | Type 2 Diabetes | E11 | 8 % | A01A, A10B | ICD10: E10, E11, E12, E13 ICPC2: T89, T90 |
| Diabetes (type1)  With/without complications | Other | E10 | 1 % | A01A, A10B | ICD10: E10, E11, E12, E13 ICPC2: T89, T90 |
| Chronic obstructive pulmonary disease | COPD | J43.x–J44.x, | 19 % | R03AC(excluding: R03AC02-04), R03AK, R03AL, R03BB, R03DA, R03DX07 | ICD10: J43, J44 ICPC2: R95 |
| Rheumatologic and musculoskeletal disorders | RMD | M05.x-M08.x, M45, M46, M30.x-M34.x, M35.0- M35.2 | 3 % | L01BA01, L04AX03, P01BA01, P01BA02, A07EC01, L04AA01,L04AA06, L04AA13, L04AD01, L04AD02, L04AK01, L04AX01, L04AB, L04AA24, L04AA51, L04AC03, L04AC05, L04AC07, L04AC07, L04AC10, L04AC13, L04AG04, L01FA03, L01XC02, L04AG04, L01FA03, LO1XC02, L04AF01-F04, H04 (>7.5 mg/day) | ICD10: M05, M06, M07, M08, M46, M45, M30, M31, M33, M34, M35 ICPC2: L88, L99 |
| Other chronic pulmonary disease | Other | J41.x, J42x, J45x, J46x, J47x, J60x-J67x, I278, I279, J684, J701, J703 | 3 % |  |  |
|  |  |  |  |  |  |
| Renal disease | Other | I12.0, I13.1, N03.2–N03.7, N05.2–N05.7, N18.x, N19.x, N25.0, Z49.0–Z49.2, Z94.0, Z99.2. NCMP: RXGD | 1 % |  |  |
| Dementia | Other | F00.x–F03.x, F05.1, G30.x, G31.1 | 1 % |  |  |
| Aids | Other | m05.x, M06.x, M31.5, M32.x–M34.x, M35.1, M35.3, M36.0 | 0.1 % |  |  |
| Peptic ulcer disease | Other | K25.x–K28.x | 1 % |  |  |
| Hemiplegia | Other | G04.1, G11.4, G80.1, G80.2, G81.x, G82.x, G83.0–G83.4, G83.9 | 0.30 % |  |  |
| Mild Liver disease | Other | B18.x, K70.0–K70.3, K70.9, K71.3–K71.5, K71.7, K73.x, K74.x, K76.0, K76.2–K76.4, K76.8, K76.9, Z94.4, | 1 % |  |  |
| Mod-severe liver disesase | Other | I85.0, I85.9, I86.4, I98.2, K70.4, K71.1, K72.1, K72.9, K76.5, K76.6, K76.7. | 0.50 % |  |  |

Abbreviations: CVD: cardiovascular disease, COPD: chronic obstructive pulmonary disease, and RMD: rheumatic and musculoskeletal disorders

**Table S2** Proportion of patients receiving systemic anticancer treatment among patients diagnosed in the pre-ICI (2012–2016) and post-ICI (2017–2021) period, stratified by comorbidity groups.

|  | **pre-ICI** | **post-ICI** | **p-value** |
| --- | --- | --- | --- |
| Overall  cohort | 52 % | 57 % | p<0.001 |
| COPD | 45 % | 51 % | p=0.005 |
| CVD | 38 % | 45 % | p=0.001 |
| T2D | 43 % | 48 % | p=0.160 |
| RMD | 40 % | 50 % | p=0.022 |

Abbreviations: COPD: chronic obstructive pulmonary disease, CVD: cardiovascular disease, T2D: Type 2 diabetes and RMD: rheumatic and musculoskeletal disorders, ICI: immune checkpoint inhibitors

**Table S3** Six- and 24-month overall survival among patients with advanced NSCLC receiving systemic anticancer treatment, diagnosed during the pre-ICI (2012–2016) and post-ICI (2017–2021) periods. Survival estimates and relative increases between periods are presented, stratified by comorbidity group.

|  | 6 month Overall survival ( %) | | | 24 month overall survival (%) | | |
| --- | --- | --- | --- | --- | --- | --- |
|  | pre-ICI | post-ICI | Ratio increase (postICI/preICI) | pre-ICI | post-ICI | Ratio increase (postICI/preICI) |
| Overall  cohort | 62% | 68% | 1.10 | 13% | 27% | 2.1 |
| COPD | 65% | 67% | 1.03 | 10% | 28% | 2.8 |
| CVD | 57% | 64% | 1.12 | 9% | 26% | 2.9 |
| T2D | 62% | 65% | 1.05 | 7% | 21% | 3.0 |
| RMD | 61% | 65% | 1.07 | 10% | 24% | 2.4 |

Abbreviations: CVD: cardiovascular disease, COPD: chronic obstructive pulmonary disease, T2D: Type 2 diabetes and RMD: rheumatic and musculoskeletal disorders, ICI: immune checkpoint inhibitors

**Table S4** 24 months overall survival among patients with advanced NSCLC receiving systemic anticancer treatment, stratified by treatment type

|  | 24-months overall survival (%) | | | |
| --- | --- | --- | --- | --- |
|  | Chemotherapy (pre-ICI) | Chemotherapy (post-ICI) | ICI | ICI + chemotherapy |
| All | 12% | 17% | 35% | 28% |
| COPD | 9% | 17% | 33% | 32% |
| CVD | 7% | 14% | 38% | 22% |
| T2D | 4% | 10% | 29% | 24% |
| RMD | 10% | 24 % | 23% | 20% |
|  |  |  |  |  |

Abbreviations: CVD: cardiovascular disease, COPD: chronic obstructive pulmonary disease, T2D: Type 2 diabetes and RMD: rheumatic and musculoskeletal disorders, ICI: immune checkpoint inhibitors

Supplementary figures

**Figure S1** Flow chart

**Sensitivity analysis**

A sensitivity analysis was performed on the adjusted model. The analysis is restricted to patients <80 years of age and ECOG performance status<2. The results are shown in figure S2.

**Figure S2** Risk of death from any cause among patients with a specific comorbidity vs those without that diagnosis, stratified by first-line systemic anticancer therapy. A hazard ratio of 1 indicates that the comorbidity does not increase the risk of death compared to patients without that comorbidity. All models were adjusted for sex, age, histology, education, stage and the presence of multiple (2 or more) comorbidities. Analyses related to first-line ICI treatment were also adjusted for PD-L1 expression and ECOG PS.

Abbreviations: ICI: immune check point inhibitors, CVD: cardiovascular disease, COPD: chronic obstructive pulmonary disease, T2D: Type 2 diabetes and RMD: rheumatic and musculoskeletal disorders, Mono:monotherapy, Chemo: chemotherapy
